# Ward rounds in internal medicine: Validation of an Entrustable Professional Activity (EPA) observation checklist

**DOI:** 10.3205/zma001164

**Published:** 2018-05-15

**Authors:** Valerie Schmelter, Esther März, Christian Adolf, Teresa L. Wölfel, Christian Lottspeich, Martin R. Fischer, Ralf Schmidmaier

**Affiliations:** 1 Klinikum der Universität München, Institut für Didaktik und Ausbildungsforschung in der Medizin, München, Germany; 2 Klinikum der Universität München, Medizinische Klinik und Poliklinik IV, München, Germany

**Keywords:** entrustable professional activity, EPA, internal medicine ward round, observation checklist, validation study

## Abstract

**Objectives: **Ward rounds serve a crucial daily activity in hospitals. Building on the Entrustable Professional Activity “Conducting internal medicine ward rounds” consisting of ten competencies and 25 corresponding activities, this study aims at assessing content and external validity of an observation checklist for this EPA.

**Methods: **A focus group aimed at content validation of the checklist. Five participants therefore evaluated it with respect to comprehensiveness and comprehensibility. To investigate external validity, 14 authentic ward rounds were video-taped and rated by two raters with the adapted observation checklist in terms of the appearance of certain activities in the videos.

**Results: **After some adaptions, participants of the focus group agreed on a checklist consisting of nine competencies, 25 activities and 110 examples of observable behaviours supporting content validity. External validity was studied by using the observation checklist for ratings of ward round videos. The checklist was regarded as a valuable tool to structure observation. Along with the high frequencies of observed behaviour and interrater-reliability, external validity can be assumed.

**Conclusion:** The first scientifically developed comprehensive observation checklist for the EPA *conducting a ward round in internal medicine* is presented. The checklist is a valuable tool for providing elaborated feedback in undergraduate and graduate medical education. Focussing on multi-institutional validation and the cut offs of the checklist to determine the levels of entrustment are recommended for future research.

## Zusammenfassung

**Ziel: **Visiten stellen eine zentrale tägliche Aufgabe im Krankenhausalltag dar. Auf der Basis der Entrustable Professional Activity „Durchführung von Visiten in der Inneren Medizin“, die 10 Kompetenzen und 25 dazugehörige Tätigkeiten umfasst, ist das Ziel dieser Studie die Bewertung der inhaltlichen und externen Validität der Beobachtungs-Checkliste.

**Methode: **Eine Fokusgruppendiskussion diente der inhaltlichen Validierung der Checkliste. Fünf TeilnehmerInnen bewerteten die Checkliste hinsichtlich der Vollständigkeit und Verständlichkeit. Zur Prüfung der externen Validität wurden 14 authentische Visiten aufgezeichnet. Zwei Beobachter füllten die Checkliste hinsichtlich des Auftretens bestimmter Tätigkeiten in den Videos aus.

**Ergebnisse: **Nach einigen Anpassungen bewerteten die TeilnehmerInnen der Fokusgruppe die Checkliste, die final aus neun Kompetenzen, 25 Aktivitäten und 110 Beipielen beobachtbaren Verhaltens besteht, als umfassend. Dies bestätigte die inhaltliche Validität der Checkliste. Die externe Validität wurde im Rahmen der Betrachtung von Visitenvideos untersucht. Die Checkliste wurde als wertvolles Hilfsmitel angesehen, die die Beobachtung strukturiert. Die hohe Häufigkeit beobachteter Verhaltensweisen sowie die hohe Interrater-Reliabilität bekräftigen die externe Validität der Checkliste.

**Schlussfolgerung: **Eine erste wissenschaftlich entwickelte umfassende Beobachtungs-Checkliste für die EPA “Durchführung einer Visite in der Inneren Medizin” wird dargestellt. Die Checkliste ist ein wertvolles Instrument, um elaboriertes Feedback im Medizinstudium und in der postgraduierten Medizin zu geben. Weitere Forschung sollte eine multi-institutionelle Validierung sowie die Differenzierung der Levels of Supervision in den Blick nehmen.

## 1. Background

Conducting clinical ward rounds is an important daily routine that every physician is expected to perform once he or she is graduated [1]. Ward rounds serve two purposes: First, they constitute an encounter for the planning and evaluation of patients’ treatment in which the different professions involved in patient care see the patient [2], [3]. Second, they provide an educational encounter in which clinically relevant knowledge and skills can be acquired by applying biomedical knowledge in its intended context [4]. Possible learning goals refer to physical examination, communication with both patients and the ward round team, professionalism, diagnosis, treatment and also economic and ethical considerations in patient care [4], [5], [6]. Thus, the participation in ward rounds contributes to preparing students for their future professional practice. Despite their importance, the educational potential of ward rounds often remains underutilized [7], [8]: students rarely participate in them [6] and report a lack of structured supervision when they do [4]. Beyond, empirical results hint at students’ deficits in essential ward round competences, particularly as regards the competences of reviewing charts, prescribing medications, documenting treatment plans [1], and communication with the patient and the ward round team [2]. With respect to the professional context of the ward round, students struggle in understanding both the ward round process and the responsibilities of ward round participants [7]. Several explanations are provided for these shortcomings which are related to the medical staff, such as limited formal training and teaching experience in general, but also in the ward round context specifically [7], [9] difficulties in integrating the different aims of ward rounds [5], [10] and insecurities regarding the roles of attending students in particular [11]. Some difficulties regarding ward rounds were intended to be reduced with the implementation of checklists that target the ward round process as such [12], [13] and specific ward round competences such as communication and interprofessional teamwork [2]. Prior attempts of developing checklists [2], [12], [13] incorporated only few activities and targeted only specific structural aspects of the ward rounds or focused on selected competences. Most important they lack a clear theoretical concept and were not developed on a scientific basis. Our previous study [14] was the first, that systematically explored the ten main competences for conducting a ward round. To assess these competencies content and external validation of the observation checklist was needed. 

### A description of ward round competences referring to the concept of Entrustable Professional Activities (EPA)

With our previous study [14], we conceptualized educational goals for ward rounds in the language of an Entrustable Professional Activity: “Conducting internal medicine ward rounds”. As such, an EPA is an instrument that enhances the decision whether or not a task can be entrusted to a trainee [15] by differentiating five levels of required supervision reflecting the increasing responsibility a trainee gains:

1. be present and observe,
2. act with direct supervision,
3. act with indirect supervision,
4. act without supervision, and
5. provide supervision.

In the undergraduate medical education the fifth level cannot be reached but can be attempted after medical graduation.

The EPA locates competences in the context of professional practice and clarifies the tasks and responsibilities of a trainee when conducting ward rounds. Besides, the EPA serves an elaborated and objective overview that provides a basis for feedback and assessment that can then be applied in both undergraduate and graduate medical education [11]. 

Our previous interview study explored competences of residents and summarized corresponding activities and tasks a graduated resident with ward round responsibilities should be able to carry out [14]. A total of 26 ward round experienced physicians and nurses representing the key professions involved in internal medicine ward rounds were interviewed. They represented the different specialisations in internal medicine and worked in hospitals of different care levels. The interviews focussed on all relevant ward round competences and activities to research the requirements of conducting ward rounds in internal medicine. From the interviews we identified ten competences relevant for conducting ward rounds in internal medicine:

1. communication with the patient,
2. communication with the team,
3. diagnostic analysis and therapy planning,
4. empathy,
5. leadership skills,
6. management of difficult situations and faults,
7. organization skills,
8. professionalism,
9. self-management, and
10. teaching and learning abilities.

Each competence was characterized by a set of activities to depict professional practice as detailed and comprehensible as possible. On this basis, the EPA “Conducting an internal medicine ward round” was compiled.

However, it remained unclear whether the EPA could be easily implemented as a feasible observation tool for the characteristics of ward rounds conducted at a university hospital. 

#### Aim of the presented project

For this purpose, we conducted two separate validation studies aiming for both content and external validation of the EPA observation checklist to making it useful for educational purposes on ward rounds in internal medicine and for further research.

The project was driven by two questions:

1. Does the EPA observation checklist represent a comprehensive and comprehensible overview of required ward round competences and activities?
2. Can the observation checklist be applied as comprehensive and feasible observation tool for ward rounds conducted by ward round teams?

## 2. Methods

### 2.1. Development and content validation of the EPA checklist 

#### Transferring the EPA into an observation list

To be applicable in professional practice, the content of an EPA needs to be expanded by detailed descriptions of observable behaviour and the context for those behaviours [15]. The original EPA “Conducting an internal medicine ward round”, which initially consisted of ten competences and 25 corresponding activities thus was further refined and complemented by a compilation of examples that made each of the 25 activities graspable and allowed a clear decision whether or not it was observable. Finally, a total of 110 exemplary behaviours were assigned to their corresponding activities and competences, contained in the original version of the EPA.

##### Focus group

To ensure content validity of the observation checklist, a focus group was conducted. While focus groups are often used for explorative purposes, they are also employed as a tool for (cross-) validation. Through interactions between participants, the information provided by preliminary data can, in the optimal case, be strengthened and extended [16].

##### Focus group participants

The five participants which reflected the different medical stakeholders involved in ward rounds were chosen for the focus group: four physicians working on an internal ward having work experience in both patient care and academic teaching (M=16.75; SD=11.53 years); one fourth year medical student who had already gained a substantial ward round routine build within medical studies and clerkships. 

##### Preparation for the focus group discussion within a computer-based environment

Due to the complex nature of the observation checklist, participants were given the opportunity to familiarize themselves with the content of the checklist one week ahead of the focus group. For this purpose, they were provided with access to the computer-based environment CASUS which was originally developed as learning platform for problem-based learning in medicine [17]. On the platform, participants were informed about the theoretical background of EPAs and with the development process of the EPA “Conducting an internal medicine ward round” with a short introductory section. After the introduction, each competence with its corresponding activities and examples were presented on a separate electronic card. 

##### Focus group discussion

After the preparation phase, the actual focus group took place. It was sub-divided into three parts:

1. introduction phase,
2. discussion of identified competences and activities,
3. application opportunities of the checklist.
4. Both the concept of EPAs and the development of the EPA “Conducting an internal medicine ward round” were recapitulated shortly. Participants were introduced to the observation checklist and the aim of the focus group procedure. They were further provided with the opportunity to ask questions.
5. The following discussion was guided by a pre-structured discussion guideline. Questions focused on the conceptualization and structure of the observation checklist but also on the comprehensibility and appropriateness of the examples. Participants further evaluated whether the checklist as a whole serves a proper representation of ward rounds, hence, a good basis for assessing ward round practice.
6. The final part of the focus group discussion focused on possible areas and settings of checklist application in both undergraduate and graduate education.

##### Computer-based follow-up phase 

Video and audio data of the focus group were transcribed and the contributions of each participant were summarized, critically assessed and the observation checklist was refined. To verify that the adaptions made on the basis of participants’ suggestions reflect their true intentions, participants received the revised checklist through the CASUS environment. Each participant was asked to provide feedback and, if necessary, suggest further adaptions. The checklist was finalized when all participants had no further comments. The final checklist is shown in attachment 1 .

#### 2.2. External validation of the EPA checklist 

To assess the checklist’s external validity, it was applied for the observation of authentic video-taped ward rounds conducted by seven ward round teams on internal medicine wards at our university hospital. 

##### Preparation phase and setting

To avoid strain for real patients, standardized patients were used. Two standardized patient cases were developed by two experienced internists (CL and RS):

1. the first case described a patient experiencing anaphylaxis and
2. the second case comprised a patient with fever of unknown origin.

The cases reflected typical diseases in internal medicine and were assumed to be solvable by internists, independent of their subspecialisation. The cases intentionally differed in their complexity to reflect the variance of ward rounds as depending on the severity of a disease. The standardized patients were trained using a detailed role script. 

A standardized final year medical student joined each ward round team on their daily ward and provided a short patient presentation according to a detailed script ahead of the consultation of the standardized patients.

##### Acquisition of video data

Seven routine ward round teams from different internal medicine wards of our university hospital participated (see Table 1 (Tab. 1)). To increase ecologic validity, the team composition and the number of team members reflected the respective daily ward practice. Some ward rounds were conducted by junior residents, others by senior residents accompanied by junior residents. Two ward round teams were additionally attended by a nurse. Table 1 (Tab. 1) shows the composition of the seven ward round teams. Along with the Declaration of Helsinki, participation based on informed consent and the ethic committee of the medical faculty approved the study. Each ward round was videotaped with two cameras (one in front of and one inside the patient’s room) including all phases of the round (preparation, preliminary discussion of a patient, patient consultation and post-processing). The teams did not receive any instructions on how to perform the ward round, but were asked to conduct the ward in the usual manner. 

##### External validation of the checklist through ratings of the videos

In a first step, two raters (VS and CA) familiarized themselves with the coding procedure referring to the checklist and the belonging coding instructions. For test evaluation, two raters independently watched two ward round videos and filled out the observation checklist sheet (see attachment 1 ) along the coding instructions. The ratings were compared, discrepancies were discussed and descriptions in the observation list were refined. The two raters independently watched all videotaped patient encounters during the ward rounds and rated all of them according to the activities listed in the EPA checklist. 

Whenever an exemplary behaviour characteristic for an activity was observed, the corresponding activity was considered as being observed. Accordingly, whenever a rater ticked one activity characteristic for a competence, the respective competence was considered as being observed. Whenever a competence was not required, the rater ticked the option ‘not applicable’ in the checklist. When a competency was required but not observable in a specific situation, no items were ticked. All the other exemplary behaviours listed below the not required competence were excluded from the further comparison of the ratings. Interrater-reliability was calculated across all videos resulting in a Cohen’s Kappa coefficient of 0.73 which is regarded as a sustainable agreement [18]. 

## 3. Results

### 3.1. Content validation of the EPA observation checklist through the focus group discussion

The focus group discussion lasted 118 minutes and enabled a lively discussion in which each participant was involved and contributed his or her opinion and experience. The participants approved the observation checklist as a useful tool not only for elaborated summative and formative feedback and assessment but also for stimulating individuals’ learning and self-reflection.

Participants agreed on nine of the initial competences which are appropriate to picture a typical ward and the corresponding affordances at our university hospital:

1. communication with the patient,
2. communication with the team and leadership skills,
3. diagnostic process and therapy planning,
4. empathy,
5. management of difficult situation and faults and leadership skills,
6. organization skills,
7. professionalism,
8. self-management, and
9. teaching and learning abilities.

The participants of the focus group discussion regarded the leadership skills as an important area of competence which cannot be treated separately but should be assessed in light of other ward round competences. Competences, and hence, activities, were mainly linked to team and patient communication and the management of difficult situations and faults. Participants pointed out that variations in the demonstrated competences may arise depending on the ward round goals (patient care vs. teaching), patient characteristics (e.g. personality, severity of disease) and composition of the ward round team (number and profession of participants).

The participants also made adaptions in the wording and description of some activities. They agreed that the high grade of detail of the provided checklist facilitates a better understanding of the relevant competences and activities and increases the applicability as a tool for feedback and assessment. However, they indicated that this complexity may impede the intuitive application on the round. 

In the follow-up phase the revised version of the checklist was presented to all participants using the CASUS tool. Regarding each revised competence the respective activities and exemplary behaviours were presented. Individuals had to indicate whether or not he or she agrees on this final version. In case of disagreement, he or she was asked to provide an explanation for the respective reason. The final version of the EPA checklist is shown in attachment 1 . 

#### 3.2. External validation of the EPA checklist through observation of standardized real life ward round videos 

With respect to generalizability a multi-institutional validation in a real life setting would be desirable. For scientific reasons however a standardization of the work place scenario was needed. To assess the external validity of the checklist, we thus chose to videotape real life ward rounds at different hospitals on different wards with different ward round teams. An overview on the duration of the video-taped ward rounds can be seen in Table 2 (Tab. 2).

The research question was, to which extent the checklist is suitable to structure the observation of ward rounds and whether the checklist aligns with typical ward rounds. The observation checklist was applied successfully and 488 behavior patterns representing the 25 ward round activities respective nine competences were coded. Six of the competencies were observed in each ward round video irrespective of the ward round team and case characteristics. Two competences – management of difficult situations and leadership skills, self-management – and their corresponding activities could not be observed in any ward round video. Besides, three activities (trainee assigns tasks, trainee documents the patient’s medical condition, new findings and planned procedure, trainee recognizes the necessity of empathic acting in the physician-patient interaction) could only be observed in some videos. Attachment 2 provides a comprehensive overview on the frequencies of codings. The raters evaluated the checklist as a valuable guide for observation. Also, the coding rules allowed reliability of ratings. 

## 4. Discussion and conclusion

Conducting ward rounds is a key responsibility of a physician once he or she is graduated. Nevertheless, research highlights that both physicians and students struggle in conducting and understanding the purpose of ward rounds. With our previous study [14], we developed an EPA “Conducting internal medicine ward rounds” that reflects the broad range of competencies and relating activities a physician should be able to show after graduation. To assess content and external validity of the observation checklist emerging from this EPA, we conducted two separate validation studies.

### Content and external validity of the observation checklist

Participants of the focus group reached consensus that the initially identified competencies picture ward round practice at our university hospital. However, participants highlighted that leadership skills should not be regarded in isolation but be assessed in light of other areas of competency. After some adaptions in the wording and descriptions of activities, participants agreed that the activities and examples for these activities included in the observation checklist are relevant for and comprehensive with respect to describing ward round competence. We conclude that content validity is given. External validity was studied by using the observation checklist for ratings of 14 authentic ward rounds performed by seven internal medicine ward round teams. Raters indicated that the observation checklist provided a valuable tool for structuring observation of the ward round process and that the activities and examples align with professional practice. Along with this insight, the high frequencies of observed behavior associated with the nine ward round competencies support external validity.

Difficulties in the application of the observation checklist

Nevertheless, results demonstrate that activities representing the competencies *management of difficult situations and leadership skills* and *self-management* could not be observed. Also activities tied to *communication with the team* and *leadership skills, diagnostic analysis and therapy planning* were only observed in some videos. It is likely, that the design of the study impeded the appearance of certain activities. We used standardized cases that ensured comparability of data. However, the cases did not involve critical situations that triggered the handling of disturbances or faults. Besides, we only videotaped parts of the ward round process at one single occasion. Videos included each a patient consultation and the associated preliminary discussion and debriefing. For practical reasons, the preparation and post-processing of the ward rounds were not subject of the video even though they would have provided additional information. To facilitate grounded entrustment decisions on a trainee’s performance, we therefore recommend to observe trainees across multiple ward round occasions enabling him or her to deal with variations in ward rounds and patient characteristics and to also show behavior that is not necessarily relevant for every ward round encounter [19], [20]. For ecological reasons, we did not manipulate the composition of the ward round team, nor did we set a time frame for the duration of the ward rounds. The variations in the team composition and the durations of the ward rounds possibly affected our results.

In addition to these design-related limitations, some activities and examples for them were not observable as they refer to an individual’s cognitive processes. Per definition, these activities cannot find their way into the observational checklist [21]. Due to their importance, these competencies should not be neglected but be addressed through other formats. Incorporating structured after-round feedback processes targeting non-observable aspects or enhancing observation data with indirect measurement tools such as questionnaires on satisfaction with the ward round process [22] or self-reflection [23] might contribute to entrustment decisions. Also the duration and interruptions of rounds can serve as complementary indicator for the efficiency of rounds [2], [24] and for entrustment decisions. 

#### Decisions on the Level of Supervision (LoS) 

Entrustment decisions strive to inform about a trainee’s ability to master a professional task and to determine the level of required supervision. These decisions are complex in nature and are shaped by several factors such as the trainee, the supervisor, the context, the task and the relationship between trainee and supervisors [15], [25]. To our knowledge, there exists no gold standard of how and when to decide whether or not a task can be entrusted to a trainee. Instead these decisions are based on formal criteria such as graduation or information gained through self-assessment and assessment of the supervisor [26], [27], [28] while acknowledging that these assessments do not necessarily suit professional practice. Clear delimitations between the different levels of required supervision are barely found. Likewise, our observation checklist does not allow a clear threshold between the five levels of required supervision. However, we understand it as a tool that structures observation of ward round performance that contributes to entrustment decisions. Using the checklist for formative assessment at multiple occasions in the course of medical education and professional development, it potentially provides significant stimuli for individual learning for both undergraduate and graduate medical education.

##### Limitations and further directions

This study examines the validity of an observation checklist developed for student education and educational research. By testing a rather theoretical model (EPA) in a practical setting (observational checklist) it constitutes an improvement of already existing checklists in the literature and builds a bridge between theory and practice.

The second part of the study addresses the external validation. We chose to standardize the patient cases and to perform checklist rating upon videotapes and not by attendance of the raters during the real-life ward rounds, which might have influenced the behavior of the teams. This enabled us to assess seven different teams under standardized conditions and revealed high quality data. However, in terms of generalizability and with the goal to determine thresholds for the different levels of entrustment, the number of scenarios is far too low. This issue is recommended to be addressed by upcoming studies. 

The presented observation checklist features a comprehensive compilation of ward round competences and their corresponding activities that were translated into graspable examples for observable behavior. It goes beyond prior attempts [1], [2] that incorporated fewer activities and targeted only specific aspects of the ward round. With our data, we could demonstrate both content and external validity of the checklist and suggest an implementation of the checklist for both undergraduate and graduate medical education. We regard the observation checklist as a valuable tool for evidence-based assessment and elaborate formative and summative feedback that enhances individuals’ professional development. 

As pointed out by the focus group, with its 25 activities and 85 exemplary facets of behavior, the observation checklist is quite extensive and therefore bears the potential to impede the intuitive application in the course of the ward round. Hence, we encourage both trainees and supervisors to familiarize themselves with the checklist before application to maximize educational outcomes. Moreover, as pointed out before, we recommend the implementation of after-round feedback processes that can be enriched with additional information sources (e.g. questionnaires) to enhance reflection on the ward round procedure and to amend learning and professional development. As stressed out by prior research, medical staff does not necessarily possess appropriate teaching competences [29]. Specific training on how to use the observation checklists can be reasonable. 

We provide a science based observation checklist for the EPA “Conducting an internal medicine ward round”. The checklist was furthermore shown to be a feasible instrument for assessing ward round performance. The implementation of the observation checklist in both the workplace and medical education are desired next steps. Accompanying research should tackle the instrument’s reliability for assessment purposes and its feasibility in the workplace.

## Competing interests

The authors declare that they have no competing interests. 

## Supplementary Material

Checklist including competencies, associates activities and examples (* marked exemplary behaviours were not observed in any of the 14 ward round scenarios)

Frequencies of competences and activities observed in the ward round videos (across both cases)

## Figures and Tables

**Table 1:**
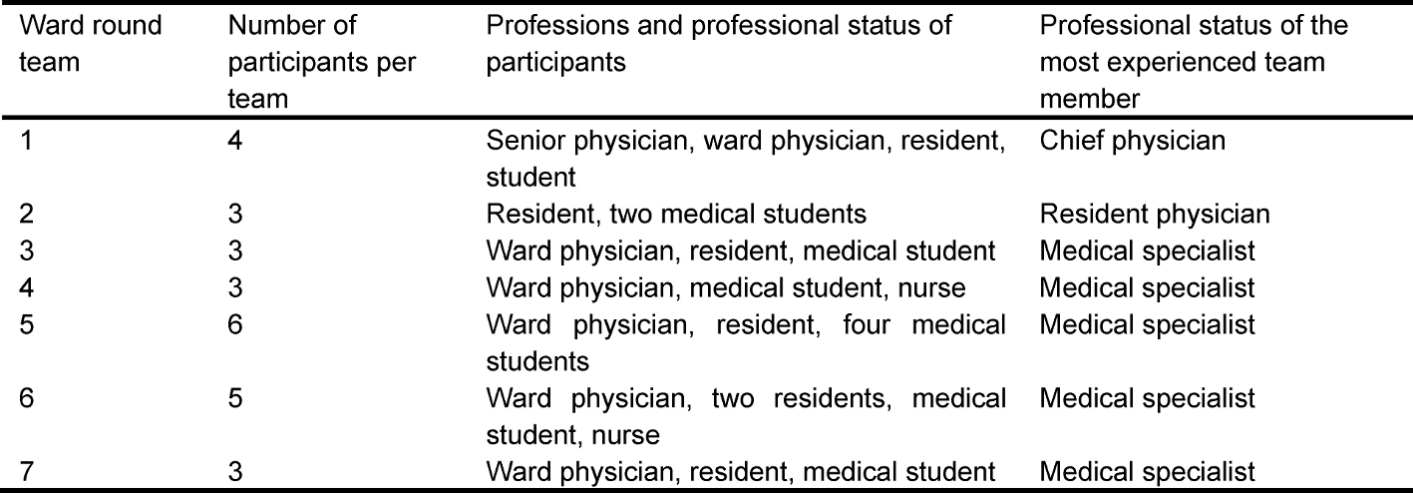
Characteristics of the participating ward round teams (excluding the standardized student)

**Table 2:**
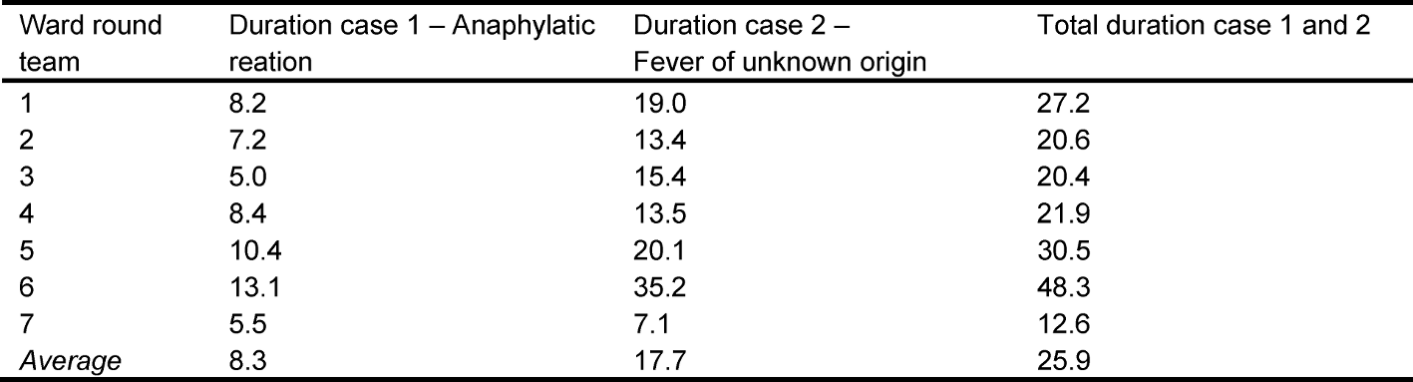
Duration of the ward round videos for case 1 and 2 and across both cases in minutes
