# Supplementary material for: Ward rounds in internal medicine: Validation of an Entrustable Professional Activity (EPA) observation checklist
Source: GMS J Med Educ. 2018 May 15;35(2):Doc17. doi: 10.3205/zma001164 (PMC6022584; doi:10.3205/zma001164)
Supplement: Checklist including competencies, associates activities and examples (* marked exemplary behaviours were not observed in any of the 14 ward round scenarios) [file JME-35-17-s-001.pdf]

Attachment 1: Checklist including competencies, associates activities and examples (* marked exemplary behaviours were not observed in any of the 14 ward round scenarios)

1. Communication with the patient	Not applicable ☐	Number of observed examples: ____

a) Trainee collects information from the patient

when the trainee sees the patient for the first time:

Trainee

- □ introduces him/herself to the patient and explains his/her own function in the hospital (e.g. junior doctor)
- □ starts the communication with the patient by addressing a non-medical topic which might concern the patient („ice-breaker“)*
- □ poses open questions (which e.g. concern the patient’s condition and needs)
- □ takes the patient’s history
- □ encourages the patient to ask questions or to add any missing information

when the trainee has already seen the patient several times:

Trainee

- □ starts the communication with the patient by addressing a non-medical topic which might concern the patient („ice-breaker“)*
- □ poses open questions (which e.g. concern the patient’s condition or needs)
- □ encourages the patient to ask questions or to add any missing information

b) Trainee informs the patient about further treatment/discharge from hospital

when the patient’s treatment in the hospital continues:

Trainee

- □ gives reasons why the planned investigation/treatment is necessary
- □ informs the patient about the advantages/disadvantages/ adverse effects of the treatment and outlines possible alternatives
- □ informs the patient about the test results
- □ gives the patient an overview of the treatment plan (e.g. all upcoming investigations)
- □ explains the timeframe of the planned procedure

for all the points mentioned above:

- □ avoids technical terms, if possible, or explains them to the patient when they are difficult to understand

when the patient will be discharged from the hospital:

Trainee

- □ gives reasons why the planned investigation/ treatment is necessary
- □ informs the patient about the discharge from hospital and explains a possible necessary follow-up treatment to the patient
- □ gives advice to the patient on how to behave after his/her discharge

for all the points mentioned above:

- □ avoids technical terms, if possible, or explains them to the patient when they are difficult to understand

c) Trainee adapts the given information to the patient’s needs

Trainee

- □ informs the patient according to his/her need for additional information
- □ informs the patient that he/she can obtain additional information at any time*

d) Trainee encourages the patient to ask questions

Trainee

- □ asks the patient whether he/she has questions and tells him/her that there is enough time for answering them
- □ lets the patient summarize the given information to make sure he/she understood him/her properly*
- □ gives information without using technical terms

e) Trainee focuses on relevant topics in the physician-patient talk
Trainee
- ◻ addresses only topics which are relevant for the ward round
- ◻ mentions a fixed time when physician and patient can have a longer conversation (e.g. about the next investigation)*

**2. Communication with the team and leadership skills**	Not applicable ◻	Number of observed examples: ____

a) Trainee collects information from the team
Trainee
- ◻ introduces the patient to the team (or lets a team member introduce the patient)
- ◻ asks the team members about their evaluation of the patient's current state of health and exchanges views with attendant physicians
- ◻ reads and interprets the nurse's or physician's documentation and discusses the gained information with the team members

b) Trainee exchanges views with the team before and after the ward round/each patient
Trainee
- ◻ provides all team members with the same information (e.g. explains necessary background information to the students so that they are able to follow the ward round)
- ◻ agrees with the attendant physicians who will lead the ward round in the patient's room and what should be discussed with the patient
- ◻ exchanges views with attendant team members before going into the patient's room
- ◻ exchanges views with attendant team members again after having seen the patient and having talked to him
- ◻ discusses the patient's level of information and the patient's need for information with the team

c) Trainee assigns tasks
Trainee
- ◻ assigns tasks that should be done at the present day to every team member
- ◻ makes sure whether the team members have carried out the tasks carefully which were assigned to them in the last ward rounds*

**3. Diagnostic analysis and therapy planning**	Not applicable ◻	Number of observed examples: ____

a) Trainee constitutes medical patient data
Trainee
- ◻ presents the file with the patient's laboratory findings to the team or shows the printed version of this document to the team members
- ◻ summarizes previous findings in the patient case for the whole team
- ◻ asks team members whether there are new investigation findings
- ◻ gathers additional information (e.g. in terms of previous medical reports) in case of inconclusive findings

b) Trainee conducts a focused physical examination

when an additional physical examination is necessary:
Trainee
- ◻ informs the patient about the examination (e.g. auscultation)

when another physical examination is not necessary:
Trainee
- ◻ informs the patient why another physical examination is not necessary (e.g. because nothing

□ provides a comfortable situation during the examination (e.g. keeps the patient's privacy)
□ instructs the patient properly how to act during the examination (e.g. tells the patient to continue breathing)
□ asks the patient whether it hurts while being examined
□ provides the patient with information on findings from examinations

has changed in the laboratory findings; the patient's condition has not changed)*

c) Trainee plans further treatment involving the patient in the decision-making process

when it is planned to continue the patient's treatment in the hospital:
Trainee
□ obtains the patient's informed consent before making a decision about the further treatment according to the patient's need

when it is planned to discharge the patient from the hospital:
Trainee
□ decides whether or not a discharge from hospital is possible while including the patient

optionally (depending on the patient case):
□ involves the legal guardian in the decision-making process when the patient cannot decide for him/herself any more*
□ makes sure that the patient will be cared for well at home if the patient cannot take care of himself*

d) Trainee documents the patients' medical condition, new finding, planned procedure

Trainee
□ takes notes of patient's medical conditions*
□ documents the findings from physical examination*
□ takes notes of the procedure planned for the following days
□ documents reasons for the decisions he made (e.g. when the decision has been changed based on previous medical reports)*
□ writes down which tasks have to be carried out this day
□ frames a case representation (SOAP-Note)*

**4. Empathy**	Not applicable □	Number of observed examples: ____

a) Trainee recognizes the necessity of empathic acting in the physician-patient interaction

Trainee
□ asks the patient actively whether he/she has concerns and takes extra time for the patient in the physician-patient talk*
□ talks with the patient about positive emotions and shares these feelings with the whole team (e.g. the photo of one of the patient's relatives next to his/her bed which sustains the patient and makes him/her happy)*
□ keeps eye contact with the patient or/and proceeds to the same height as the patient (e.g. by sitting on a chair next to the patient's bed)
□ shows appropriate body language towards the patient
□ encourages the patient to satisfy the patient's fears before the upcoming procedure
□ talks about his/her own feelings and the feelings of the whole team in front of the patient's room after having seen the patient during the ward round*

**5. Management of difficult situations and faults and leadership-skills**	Not applicable □	Number of observed examples: ____

a) Trainee is able to deal with interruptions
Trainee
- ◻ obtains information about the relevance of the interruptions and decides whether or not prompt action is necessary

b) Trainee is able to deal with faults
Trainee
- ◻ accepts hints of eventual faults and follows up these hints
- ◻ informs the patient about the occurrence of mistakes
- ◻ talks openly to the patient about the mistake that has been made
- ◻ informs the patient which consequences the mistake might bring along
- ◻ moderates the discussion to avoid faults in the future
- ◻ establishes the cause of the fault

c) Trainee is able to deal with conflicts in the team or with the patient
Trainee
- ◻ talks about the conflicts with all team members who were involved in the conflict (e.g. in front of the patient's room)
- ◻ appreciates all team members
- ◻ tells the patient that his/her person and his/her concerns will be taken seriously
- ◻ moderates the discussion about the conflict in a solution-oriented way is mindful of sticking to feedback rules

**6. Organization competence**	Not applicable ◻	Number of observed examples: ____

a) Trainee leads a well-structured ward round
Trainee
- ◻ sets a time frame in which the ward round takes place and is mindful of staying in schedule (e.g. 9.00 to 11.00 o' clock every day)*
- ◻ prepares the ward round by looking over all patients' files and possible new findings
- ◻ avoids interruptions (e.g. by turning off the cellphone, handing off the pager, briefing the team that interruptions are not allowed)*
- ◻ follows a standardized procedure inside and outside the patients' room

b) Trainee adapts duration of ward round to the patient and to events of the day
Trainee
- ◻ answers all questions the patient poses when the patient has an increased need for information and when the events of the day allow the required additional time
- ◻ tells the whole team and patient that extra time is necessary (when the physician-patient talk takes up more time)*

c) Trainee utilizes adequate tools
Trainee
- ◻ makes sure that someone takes notes of planned procedures at the present day
- ◻ focuses on shareable sources of information (e.g. notebook, patients' files etc.)

**7. Professionalism**	Not applicable ◻	Number of observed examples: ____

a) Trainee ensures reliable behaviour towards the ward round team and the patient
Trainee
- ◻ is loyal and authentic towards the team and the patient and keeps promises he/she made
- ◻ informs the team or the patient in time if it is not possible for him/her to keep the promise*
- ◻ acts collegially towards the team
- ◻ is on spot when the team or the patient needs help/support*

b) Trainee ensures a respectful physician-patient contact
Trainee
- ◻ protects patient's privacy
- ◻ respects the decisions the patient made

□ meets the patient eye to eye
□ talks directly to the patient and not about the patient in the third person

**8. Self-management**	Not applicable □	Number of observed examples: ____

a) Trainee acts when he/she reaches his professional and personal limits
Trainee
□ recognizes his/her professional or personal limits
□ asks for support (e.g. by calling a more experienced colleague for help) when he/she reaches his/her professional limits
□ asks team colleagues for help, when he/she has a conflict with the patient and reaches his personal limits
□ admits when he/she does not know certain aspects and promises to provide the missing information to the patient once he/she has informed him/herself

b) Trainee stays calm and behaves professionally in difficult situations
Trainee
□ stays calm in conflict situations and is still polite to the patient
□ is able to end an argument with the patient even though a solution for the conflict cannot be found
□ sympathizes with the patient's needs and anger but does not apologize for things he/she is not responsible for
□ carries out tasks reliably and correctly even though he/she has to deal with a difficult situation

**9. Teaching and learning abilities**	Not applicable □	Number of observed examples: ____

a) Trainee allows attendance of students and involves them in the ward round
Trainee
□ asks the patient for permission for students to attend the ward round and to be informed about his/her case (when the students attend the ward round for the first time)*
□ involves the students in the ward round process and lets them overtake an active role during the ward round
□ lets the students undertake a task in the ward round
□ poses a question to the students and answers the students' questions
□ provides feedback to the students

b) Trainee discusses patient cases with students to transfer medical knowledge
Trainee
□ lets students introduce the patient's case
□ provides information on the patient case, encourages students to think about the case and dissolves the case afterwards (e.g. the final diagnosis, treatment plan etc.)
□ lets the students summarize the information they gained during the discussion of the patient case at the present day
□ asks the students whether they could achieve a learning goal*
□ verbalizes his/her own reasoning to let the students participate in his/her thoughts
□ discusses the patient's clinical symptoms, disorders and possible differential diagnosis with the students
□ gives the students a chance to put the given feedback into practice*
□ gives the students an instruction for research or preparation for the next time*
