# Supplementary material for: Ward rounds in internal medicine: Validation of an Entrustable Professional Activity (EPA) observation checklist
Source: GMS J Med Educ. 2018 May 15;35(2):Doc17. doi: 10.3205/zma001164 (PMC6022584; doi:10.3205/zma001164)
Supplement: Frequencies of competences and activities observed in the ward round videos (across both cases) [file JME-35-17-s-002.pdf]

Attachment 2: Frequencies of competences and activities observed in the ward round videos (across both cases)

		Ward round team							
Competence	Activity	1	2	3	4	5	6	7	*Total*
Communication with the patient	Trainee collects information from the patient	6	8	6	7	7	7	6	47
	Trainee informs the patient about further treatment / discharge	10	11	9	6	7	11	6	60
	Trainee adapts the given information to the patient's needs	2	2	2	1	2	2	2	13
	Trainee encourages the patient to ask questions	2	4	-	3	4	3	2	18
	Trainee focuses on relevant topics in the physician-patient talk	1	2	2	2	2	2	2	13
Communication with the team and leadership skills	Trainee collects information from the team	2	4	2	3	4	2	3	20
	Trainee exchanges views with the team befort and after the ward round / each patient	8	6	3	6	6	8	4	41
	Trainee assigns tasks	2	-	-	2	2	-	-	6
Diagnostic analysis and therapy planning	Trainee constitutes medical patient data	3	6	6	3	2	4	4	
	Trainee conducts a focused physical examination	6	-	8	8	3	6	3	34
	Trainee plans further treatment involving the patient in the decision-making process	3	2	2	2	2	3	2	16
	Trainee documents the patient's medical condition, new findings, planned procedure	2	-	-	-	-	3	-	5
Empathy	Trainee recognizes the necessity of empathic acting in the physician-patient interaction	6	6	-	3	4	5	-	24
Management of difficult situations and leadership skills	*Trainee is able to deal with interruptions*	-	-	-	-	-	-	-	-
	Trainee is able to deal with a fault	-	-	-	-	-	-	-	-
	Trainee is able to deal with conflicts in the team or with the patient	-	-	-	-	-	-	-	-
Organization competence	Trainee leads a well-structured ward round	4	3	1	2	2	4	1	17

	Trainee adapts duration of the ward round to the patient and to events of the day	2	2	1	2	2	2	2	13
	Trainee uses adequate tools	2	2	1	2	4	2	2	15
Professionalism	Trainee ensures reliable behavior toward the ward round team and the patient	2	2	-	2	2	4	1	13
	Trainee ensures a respectful physician-patient contact	8	6	2	6	6	7	3	38
Self-management	*Trainee acts when he reaches his professional and personal limits*	-	-	-	-	-	-	-	-
	Trainee stays calm and behaves professionally in difficult situations	-	-	-	-	-	-	-	-
Teaching and learning abilities	Trainee allows attendance of students and involves them in the ward round	7	3	2	6	7	6	4	35
	Trainee discusses patient cases with students to transfer medical knowledge	7	3	4	2	8	3	2	29
Total		85	72	51	68	76	86	50	488

Note: Competences and activities that could not be observed are written in italics.
